# Genetic Deletion and Pharmacological Inhibition of PI3K***γ*** Reduces Neutrophilic Airway Inflammation and Lung Damage in Mice with Cystic Fibrosis-Like Lung Disease

**DOI:** 10.1155/2015/545417

**Published:** 2015-06-21

**Authors:** Maria Galluzzo, Elisa Ciraolo, Monica Lucattelli, Eriola Hoxha, Martina Ulrich, Carlo Cosimo Campa, Giuseppe Lungarella, Gerd Doring, Zhe Zhou-Suckow, Marcus Mall, Emilio Hirsch, Virginia De Rose

**Affiliations:** ^1^Department of Clinical and Biological Sciences, University of Torino, A.O.U. S.Luigi Gonzaga, Regione Gonzole 10, Orbassano, 10043 Turin, Italy; ^2^Department of Molecular Biotechnology and Health Sciences, Center for Molecular Biotechnology, University of Torino, Via Nizza 52, 10126 Turin, Italy; ^3^Department of Physiopathology, Experimental Medicine, and Public Health, University of Siena, 53100 Siena, Italy; ^4^Department of Neuroscience, University of Torino, 10126 Turin, Italy; ^5^Institute of Medical Microbiology and Hygiene, University of Tübingen, 72074 Tübingen, Germany; ^6^Department of Translational Pulmonology, Translational Lung Research Center Heidelberg (TLRC), German Center for Lung Research (DZL), University of Heidelberg, 69120 Heidelberg, Germany

## Abstract

*Purpose*. Neutrophil-dominated airway inflammation is a key feature of progressive lung damage in cystic fibrosis (CF). Thus, reducing airway inflammation is a major goal to prevent lung damage in CF. However, current anti-inflammatory drugs have shown several limits. PI3K*γ* plays a pivotal role in leukocyte recruitment and activation; in the present study we determined the effects of genetic deletion and pharmacologic inhibition of PI3K*γ* on airway inflammation and structural lung damage in a mouse model of CF lung disease. *Methods*. *β*ENaC overexpressing mice (*β*ENaC-Tg) were backcrossed with PI3K*γ*-deficient (PI3K*γ* ^KO^) mice. Tissue damage was assessed by histology and morphometry and inflammatory cell number was evaluated in bronchoalveolar lavage fluid (BALF). Furthermore, we assessed the effect of a specific PI3K*γ* inhibitor (AS-605240) on inflammatory cell number in BALF. *Results*. Genetic deletion of PI3K*γ* decreased neutrophil numbers in BALF of PI3K*γ* ^KO^/*β*ENaC-Tg mice, and this was associated with reduced emphysematous changes. Treatment with the PI3K*γ* inhibitor AS-605240 decreased the number of neutrophils in BALF of *β*ENaC-Tg mice, reproducing the effect observed with genetic deletion of the enzyme. *Conclusions*. These results demonstrate the biological efficacy of both genetic deletion and pharmacological inhibition of PI3K*γ* in reducing chronic neutrophilic inflammation in CF-like lung disease *in vivo*.

## 1. Introduction

Cystic fibrosis (CF), the most common genetic disease in Caucasian populations, results from mutations in a single gene encoding for 1480 residues transmembrane glycoprotein, the cystic fibrosis transmembrane conductance regulator (CFTR), that regulates cAMP-mediated chloride conductance at the apical surface of secretory epithelia [1, 2]. Impaired CFTR-mediated secretion of Cl- and bicarbonate results in dehydration and acidification of the airway surface liquid, which in turn causes impaired mucociliary clearance and bacterial killing. These defects trigger a progressive lung disease characterized by airway mucus obstruction, chronic neutrophilic inflammation, bacterial infection, and structural lung damage that remains the major cause of morbidity and mortality in patients with CF [3].

A growing number of* in vitro* and* in vivo* studies support the notion that chronic neutrophilic inflammation with the release of damaging neutrophil products, such as neutrophil elastase, constitutes a key risk factor in early structural lung damage and lung function decline in CF [4–6]. Neutrophilic airway inflammation is augmented after onset of chronic bacterial infection with* Pseudomonas aeruginosa* and other pathogens. In this context, the inflammatory response in the CF lung is nonresolving and self-perpetuating, and a vicious cycle of neutrophilic inflammation, noxious mediator release, and overwhelmed defenses amplifies inflammation, perpetuates infection and contributes to irreversible lung damage and disease progression [7–9]. Therefore, anti-inflammatory therapy, combined with antibiotic therapy, appears crucial to prevent chronic lung damage. However, traditional therapeutic strategies, as well as more recently studied anti-inflammatory drugs, have shown several limitations and limited clinical benefit [8–10]. Clearly, novel approaches have to be undertaken to provide effective anti-inflammatory therapy to CF patients. One possibility is to interfere with leukocyte trafficking into CF airways. Trafficking of leukocytes is controlled by chemotactic factors which bind to heterotrimeric G-protein-coupled receptors (GPCR) and trigger a complex set of signaling pathways inside the cell involving the generation of second messengers like phosphoinositides. Phosphoinositides are substrates of the phosphoinositide 3-kinases (PI3Ks), enzymes that catalyze the phosphorylation of the phosphatidylinositol at the 3rd position of the inositol ring. PI3Ks modulate a wide number of cellular functions such as proliferation and survival, cytoskeletal remodeling, and membrane trafficking and represent important mediators in the signaling cascade leading to the initiation of the inflammatory response [11–14]. PI3Ks can be divided in three classes (I, II, and III) based on their biochemical properties. Leukocytes express all four known isoforms of class I PI3Ks, namely, PI3K *α*, *β*, *δ*, and *γ* [14]; nonetheless PI3K*γ* plays a fundamental role in leukocyte migration and function by acting as a chemokine sensor and regulating neutrophil oxidative burst, T cell proliferation, and mast degranulation. We therefore hypothesized that PI3K*γ* plays a pivotal role in mediating leukocyte recruitment and activation and may thus represent a potential target for anti-inflammatory treatment to reduce neutrophilic airway inflammation and lung damage in CF. To test this hypothesis, we used transgenic mice with airway-specific overexpression of the epithelial Na^+^ channel (ENaC) and determined the effects of genetic deletion and pharmacologic inhibition of PI3K*γ* [15–17].

## 2. Materials and Methods

### 2.1. Mice

PI3K*γ* ^WT^/*β*ENaC-Tg (*β*ENaC-Tg) [15–18] and PI3K*γ-*deficient (PI3K*γ* ^KO^, Harlan, Italy) mice on the C57BL/6 background were intercrossed to generate *β*ENaC-Tg/PI3K*γ* ^KO^ mice. All experiments were performed in 7- to 8-week-old adult mice. *β*ENaC-Tg, PI3K*γ* ^KO^, PI3K*γ* ^KO^/*β*ENaC-Tg, and wild-type (PI3K*γ* ^WT^) mice were housed in a pathogen-free animal facility at the Istituto per la Ricerca e la Cura del Cancro, University of Turin, in accordance with the Institutional Animal Welfare Guidelines and Italian legislation. The animal study protocols were reviewed and approved by the Institutional Animal Ethics Committee of the Istituto per la Ricerca e la Cura del Cancro, University of Turin, Turin, Italy, and performed according to the Institutional Animal Welfare Guidelines and Italian legislation.

### 2.2. Assessment of Inflammatory Cells in Bronchoalveolar Lavage

Inflammatory cell numbers were assessed in the broncoalveolar fluid (BALF) of PI3K*γ* ^WT^ mice and of PI3K*γ* ^WT^/*β*ENaC-Tg, PI3K*γ* ^KO^, and PI3K*γ* ^KO^/*β*ENaC-Tg mice. Briefly, mice from each genotype were sacrificed and BALF was then collected by lavaging lungs in situ with 3 × 1-mL volumes of PBS. After centrifugation of the BALFs, cell pellets, in 500 *μ*L of RPMI medium, were deposited onto glass slides using a Cytospin Cytocentrifuge. Slides were then stained using the Diff-Quick system (MICROPTIC S.L., Spain) and a differential cell count was performed as previously described [19]. In addition, BALF inflammatory cells were also analyzed in mice treated with the PI3K*γ* inhibitor AS-605240 [5-(quinoxalin-6-ylmethylidene)-1,3-thiazolidine-2,4-dione] (Sigma, Germany). PI3K*γ* ^WT^ and PI3K*γ* ^WT^/*β*ENaC-Tg mice were treated once daily for 3 days with the AS-605240 by intraperitoneal injection of 10 mg/kg of the drug or vehicle (0.5% carboxymethyl cellulose, 0.25% Tween) alone.

### 2.3. Lung Histology and Morphometry

Animals of each group were sacrificed under anaesthesia with pentobarbital (60 mg/Kg) and the lungs fixed intratracheally with buffered formalin (5%) at a constant pressure of 20 cm H_2_O. Lung volume (*V*) was measured by water displacement according to Scherle [20]. Sagittal sections of each pair of lungs were cut and stained with haematoxylin/eosin. The slides were coded to prevent bias. Morphometric evaluations included determination of the average interalveolar distance (mean linear intercept: Lm) [21] and internal surface area (ISA) estimated by the Lm method at postfixation lung volume by the formula 4*V*/Lm, where *V* is the postfixation lung volume [22]. For the determination of the Lm for each pair of lungs, 40 histological fields were evaluated both vertically and horizontally. The development of goblet cell metaplasia was evaluated by periodic acid-Schiff reaction (PAS) according to standard histological protocols [23]. The total number of cells, as well as the percentage of PAS-positive cells, was determined. The number of cells in airways that demonstrated PAS staining was determined by examining eight intrapulmonary airways per section and counting at least 3,000 cells/section. Data were reported as the percentage of positive cells per total cells.

### 2.4. Statistical Analysis

Statistical analyses were performed using one-way analysis of variance. Survival curves were compared using Kaplan-Meier log rank analysis. *P* < 0.05 was considered statistically significant and “*n*” represents the number of mice in each experimental group. Data are expressed as mean ± SD.

## 3. Results

### 3.1. Genetic Deletion of PI3K*γ* Reduces Neutrophilic Airway Inflammation and Mortality in *β*ENaC-Tg Mice

**Figure 1:**
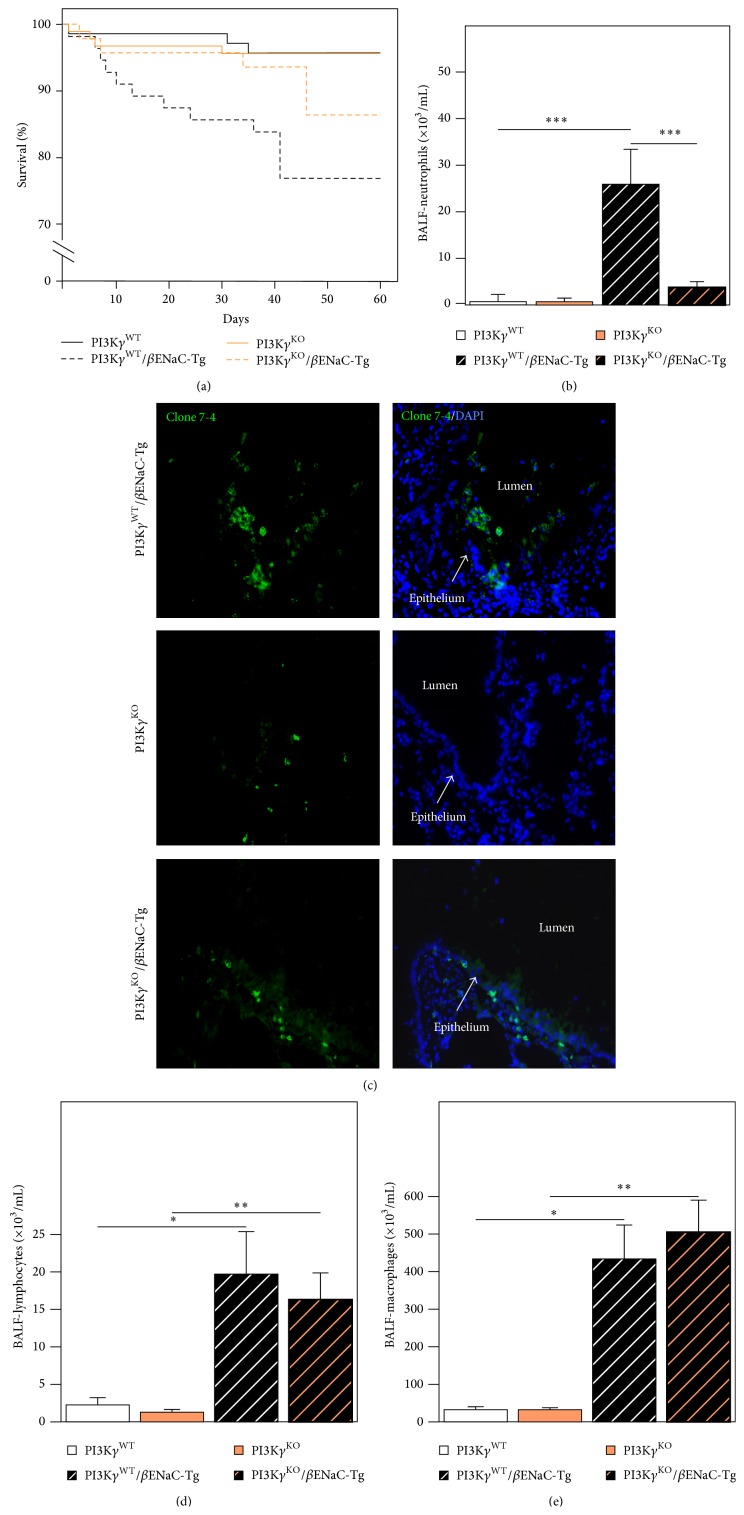
*Effect of genetic deletion of PI3Kγ on mortality and airway inflammation in βENaC-Tg mice*. (a) Survival curves for the different groups of mice studied (*P* < 0.05). (b) Neutrophil numbers were assessed in BALF of PI3K*γ* ^WT^, PI3K*γ* ^WT^/*β*ENaC-Tg, PI3K*γ* ^KO^, and PI3K*γ* ^KO^/*β*ENaC-Tg mice. Neutrophils are expressed as cell numbers per mL of BALF (*n* = 10 mice for each group). Comparison between the different groups was performed by one-way analysis of variance. ^***^ *P* < 0.001  PI3K*γ* ^WT^ *versus *PI3K*γ* ^WT^/*β*ENaC-Tg and ^***^ *P* < 0.001  PI3K*γ* ^WT^/*β*ENaC-Tg* versus *PI3K*γ* ^KO^/*β*ENaC-Tg. (c) Immunofluorescent detection of neutrophils in lung tissues of PI3K*γ* ^WT^/*β*ENaC-Tg, PI3K*γ* ^KO^, and PI3K*γ* ^KO^/*β*ENaC-Tg mice. Neutrophils were stained by using monoclonal rat antibodies to neutrophils (clone 7/4, Acris) and nuclei with DAPI. (d) Lymphocyte numbers were assessed in BALF of PI3K*γ* ^WT^, PI3K*γ* ^WT^/*β*ENaC-Tg, PI3K*γ* ^KO^, and PI3K*γ* ^KO^/*β*ENaC-Tg mice. Lymphocytes are expressed as number of cells per mL of BALF (*n* = 10 mice for each group) ^*^ *P* < 0.05  PI3K*γ* ^WT^ *versus *PI3K*γ* ^WT^/*β*ENaC-Tg and ^**^ *P* < 0.01  PI3K*γ* ^KO^ *versus *PI3K*γ* ^KO^/*β*ENaC-Tg. (e) Macrophage numbers were assessed in BALF of PI3K*γ* ^WT^, PI3K*γ* ^WT^/*β*ENaC-Tg, PI3K*γ* ^KO^, and PI3K*γ* ^KO^/*β*ENaC-Tg mice. Macrophages are expressed as number of cells per mL of BALF (*n* = 10 mice for each group). ^*^ *P* < 0.05  PI3K*γ* ^WT^ *versus *PI3K*γ* ^WT^/*β*ENaC-Tg and ^**^ *P* < 0.01  PI3K*γ* ^KO^ *versus *PI3K*γ* ^KO^/*β*ENaC-Tg.

As observed in previous studies, *β*ENaC-Tg (PI3K*γ* ^WT^/*β*ENaC-Tg; Figure 1(a)) mice on the C56BL/6J background exhibited a spontaneous mortality of ~23% [18, 24]. Deletion of PI3K*γ* had no effect on survival in wild-type mice; however, in the presence of the *β*ENaC transgene (PI3K*γ* ^KO^/*β*ENaC-Tg), PI3K*γ* loss significantly reduced the mortality by ~50%, since at 60 days the survival rate is more than 85% (*P* < 0.05, Figure 1(a)).

To determine the effect of genetic deletion of PI3K*γ* on airway inflammation, we compared inflammatory cell numbers in BAL fluid from surviving PI3K*γ* ^WT^/*β*ENaC-Tg and PI3K*γ* ^KO^/*β*ENaC-Tg mice. As expected, in homozygous PI3K*γ* ^WT^ and PI3K*γ* ^KO^ control mice, neutrophils were rarely detected in the BALF (Figure 1(b)) as well as in the airways lumen (Figure 1(c)). The number of neutrophils, in BALF and in the airways lumen, was markedly elevated in PI3K*γ* ^WT^/*β*ENaC-Tg mice (Figures 1(b) and 1(c)). On the contrary, the absence of PI3K*γ* expression in PI3K*γ* ^KO^/*β*ENaC-Tg mice led to a large reduction of neutrophil recruitment into the lung if compared to PI3K*γ* ^WT^/*β*ENaC-Tg mice (Figure 1(b)). Nonetheless, deletion of PI3K*γ* did not affect macrophage and lymphocyte recruitment as no differences were detected between PI3K*γ* ^KO^/*β*ENaC-Tg and PI3K*γ* ^WT^/*β*ENaC-Tg mice in BALF (Figures 1(d) and 1(e)).

### 3.2. Genetic Deletion of PI3K*γ* Reduces Structural Lung Damage in *β*ENaC-Tg Mice

**Figure 2:**
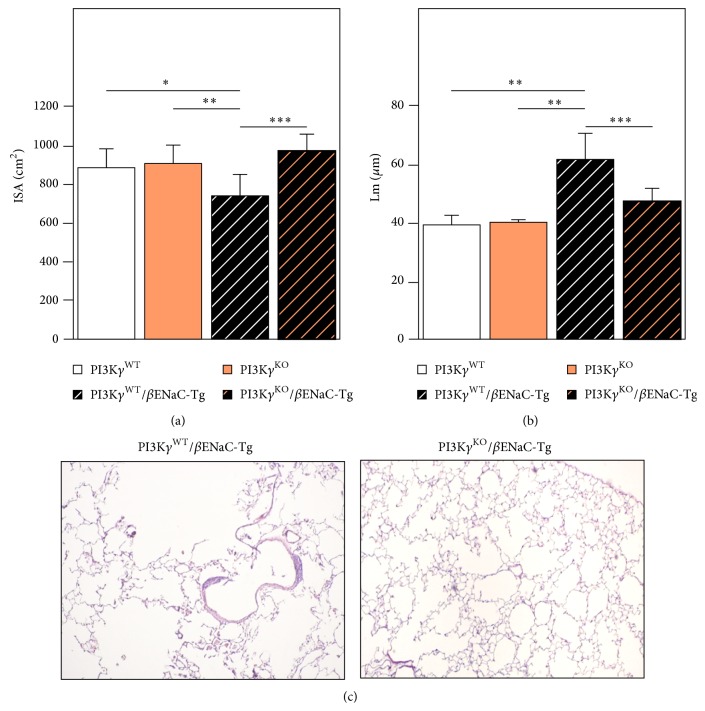
*Genetic deletion of PI3Kγ decreases emphysema in βENaC-Tg mice*. Mouse lungs were fixed in 4% formalin and embedded in paraffin and 5 *µ*m sections were stained with hematoxylin/eosin; assessment of emphysema included the internal surface area (ISA) at postfixation lung volume and the morphometric assessment of the average inter-alveolar distance (mean linear intercept: Lm). (a) ISA and (b) Lm from 8-week-old wild type (PI3K*γ* ^WT^), *β*ENaC-Tg, PI3K*γ* ^KO^, and PI3K*γ* ^KO^/*β*ENaC-Tg mice (*n* = 8–10 mice for each group). Comparison among groups was performed using one-way analysis of variance. (a) ^*^ *P* < 0.05  PI3K*γ* ^WT^ *versus *PI3K*γ* ^WT^/*β*ENaC-Tg, ^**^ *P* < 0.01  PI3K*γ* ^KO^ *versus *PI3K*γ* ^WT^/*β*ENaC-Tg, ^***^ *P* < 0.001  PI3K*γ* ^WT^/*β*ENaC-Tg* versus *PI3K*γ* ^KO^/*β*ENaC-Tg; (b) ^**^ *P* < 0.01  PI3K*γ* ^WT^ *versus *PI3K*γ* ^WT^/*β*ENaC-Tg, and *P* < 0.01  PI3K*γ* ^KO^ *versus *PI3K*γ* ^WT^/*β*ENaC-Tg, ^***^ *P* < 0.001  PI3K*γ* ^WT^/*β*ENaC-Tg* versus *PI3K*γ* ^KO^/*β*ENaC-Tg mice. (c) Representative histological sections from the lung of 8-week-old *β*ENaC-Tg mousee (left) showing evident areas of emphysema and PI3K*γ* ^KO^/*β*ENaC-Tg (right) mouse showing a focal areas of mild emphysema. Haematoxylin and eosin stain. Original magnification ×40.

Chronic inflammation, in PI3K*γ* ^WT^/*β*ENaC-Tg mice, triggers emphysema with distal airspace enlargement and alveolar destruction resulting in reduced lung tissue density and increased lung compliance [6, 17, 19]. To assess the protective effects of the genetic deletion of PI3K*γ* on emphysema-like changes in PI3K*γ* ^KO^/*β*ENaC-Tg mice, we determined the averaged interalveolar distance (mean linear intercept, Lm) and the internal surface area (ISA) estimated by the Lm method at postfixation lung volume. ISA and Lm were not altered in the lungs of controls PI3K*γ* ^WT^ and PI3K*γ* ^KO^ mice (Figures 2(a) and 2(b)), and morphological analysis showed a well-fixed normal parenchyma with normal airways (data not shown). As expected from previous studies [6, 17, 19], PI3K*γ* ^WT^/*β*ENaC-Tg mice lungs showed significant emphysematous changes (Figures 2(a)–2(c)) while the genetic deletion of PI3K*γ* in PI3K*γ* ^KO^/*β*ENaC-Tg mice resulted in a significant reduction of the degree of emphysema, as assessed by both morphometric analyses (ISA: *P* < 0.0002 versus PI3K*γ* ^WT^/*β*ENaC-Tg mice; Lm: *P* < 0.0003* versus *PI3K*γ* ^WT^/*β*ENaC-Tg mice; Figures 2(a) and 2(b)) and morphology (Figure 2(c)).

In addition to neutrophilic inflammation, goblet cell metaplasia and mucus obstruction were a common feature of the airways of adult PI3K*γ* ^WT^/*β*ENaC-Tg mice [19]. Since neutrophil products, such as neutrophil elastase, have been implicated in goblet cell metaplasia and mucin hypersecretion in CF [25, 26], we assessed the effects of genetic deletion of PI3K*γ* on goblet cell metaplasia. Goblet cells were not observed in PI3K*γ* ^WT^ and PI3K*γ* ^KO^ mice; in PI3K*γ* ^KO^/*β*ENaC-Tg mice, the goblet cell metaplasia appeared reduced compared to PI3K*γ* ^WT^/*β*ENaC-Tg mice; however, this difference was not statistically significant, based on the variability and the number of mice included in our studies (data not shown).

### 3.3. Pharmacological Inhibition of PI3K*γ* Reduces Neutrophilic Airway Inflammation in *β*ENaC-Tg Mice

**Figure 3:**
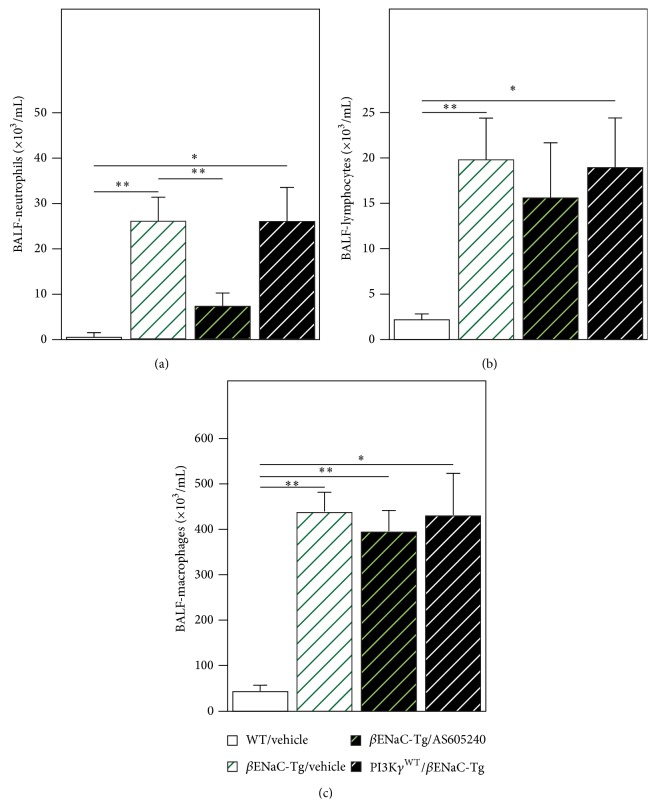
*Pharmacological inhibition of PI3Kγ decreases neutrophilic airway inflammation in βENaC-Tg mice*. Neutrophils (a), lymphocytes (b), and macrophages (c) numbers were determined in BAL fluid of control (WT/Vehicle) and *β*ENaC-Tg mice untreated or treated with the PI3K*γ* inhibitor AS-605240 (*β*ENaC-Tg/AS-605240) or with vehicle (*β*ENaC-Tg/Vehicle). Cells are expressed as number per mL of BAL fluid. (a) ^**^ *P* < 0.01 WT/Vehicle* versus β*ENaC-Tg/Vehicle, ^**^ *P* < 0.01  *β*ENaC-Tg/Vehicle* versus β*ENaC-Tg/AS-605240 and ^*^ *P* < 0.05 WT/Vehicle* versus *PI3K*γ* ^WT^/*β*ENaC-Tg. (b) ^**^ *P* < 0.01 WT/Vehicle* versus β*ENaC-Tg/Vehicle and ^*^ *P* < 0.05 WT/Vehicle* versus *PI3K*γ* ^WT^/*β*ENaC-Tg. (c). ^**^ *P* < 0.01 WT/Vehicle* versus β*ENaC-Tg/Vehicle, ^**^ *P* < 0.01 WT/Vehicle* versus β*ENaC-Tg/AS-605240, and ^*^ *P* < 0.05 WT/Vehicle* versus *PI3K*γ* ^WT^/*β*ENaC-Tg.

Next we tested effects of pharmacological inhibition of PI3K*γ* by using the inhibitor AS-605240 on airway inflammation in *β*ENaC-Tg mice. Treatment of *β*ENaC-Tg mice with AS-605240 but not with vehicle alone reduced neutrophil infiltrates in BALF of *β*ENaC-Tg mice (Figure 3(a)). In contrast, as observed in PI3K*γ* ^KO^/*β*ENaC-Tg mice, the PI3K*γ* inhibitor had no effect on the recruitment of macrophages or lymphocytes into the lung (Figures 3(b) and 3(c)).

## 4. Discussion

Progressive lung disease is the major cause of morbidity and mortality in CF and is characterized by chronic airway infection and associated airway inflammation leading to irreversible lung destruction and early death [1–3]. Accumulating evidences suggest that CFTR dysfunction impairs mucociliary clearance and bacterial killing as crucial innate defense mechanisms of the lung leading to chronic bacterial infection and nonresolving inflammation in CF airways [3]. The main feature of airway inflammation in CF is a persistent influx of neutrophils that release a variety of oxidants and granule-associated enzymes, thus contributing to the development of lung injury and to the chronicity of pulmonary infection [7–9]. Repeated episodes of exacerbation of chronic infection and inflammation occur during the natural history of the disease, further increasing the structural damage in the CF lung [27, 28]. Therefore, anti-inflammatory therapy, combined with antibiotic therapy, offers a rational approach to prevent chronic lung damage. However, current anti-inflammatory drugs have shown several limits. The use of oral corticosteroids has been limited by severe adverse effects and studies using inhaled corticosteroids in CF have not been particularly successful [8, 9]. In addition, nonsteroidal anti-inflammatory drugs, such as ibuprofen, although revealing beneficial effects in younger CF patients [29], are difficult to dose and thus are not widely used [30]. Likewise, a phase 3 study of the LTB_4_ receptor antagonist BIIL 284 had been stopped due to adverse effects in the treatment group [31]. An alternative approach to decrease chronic inflammation is to use a more targeted anti-inflammatory therapy directed at reducing neutrophil trafficking in the CF lung. In this context, class I PI3K member, PI3K*γ*, has been demonstrated to play a pivotal role in mediating leukocyte recruitment and activation into sites of inflammation [11]. Therefore PI3K*γ* may represent an innovative and appropriate target to interfere with the excessive neutrophil-mediated inflammation and damage in CF. Of note, recently developed small-molecule PI3K*γ* inhibitors were shown to be effective in suppressing joint inflammation in mouse models of rheumatoid arthritis [32]. In the present study we evaluated the effects of genetic deletion and pharmacologic inhibition of PI3K*γ* in the *β*ENaC-Tg mouse as a model of CF lung disease [15, 16, 33]. Such model phenocopies the airway surface dehydration and mucociliary dysfunction characteristic of CF airways. *β*ENaC-Tg mice develop spontaneous CF-like lung disease with early onset goblet cell metaplasia and airway mucus obstruction, reduced bacterial clearance, and chronic neutrophilic inflammation triggering emphysema-like structural lung damage [15, 17, 34, 35]. Genetic deletion of PI3K*γ* resulted in decreased neutrophil numbers in BALF of PI3K*γ* ^KO^/*β*ENaC-Tg mice, and reduced neutrophilia was associated with reduced emphysematous changes in these mice. Taken together, these data support an important role of PI3K*γ* for transmigration of neutrophils from the blood into the airway lumen and a crucial role of neutrophilic airway inflammation in the* in vivo* pathogenesis of lung damage. Several leukocyte-derived proteases including neutrophil elastase have been shown to cause emphysema in mice [36–38]. Furthermore, previous studies demonstrated that overexpression of several proinflammatory mediators in genetically modified mice induces an imbalance in the pulmonary protease/antiprotease system and emphysema in these mice [39, 40]. Thus, it is likely that neutrophil-dominated chronic pulmonary inflammation and the disruption of protease/antiprotease balance contribute to the development of emphysema in PI3K*γ* ^WT^/*β*ENaC-Tg mice. Neutrophil elastase (NE) is the major product of activated neutrophils and has been implicated in the pathogenesis of key features of CF lung disease, such as chronic airway inflammation, mucus hypersecretion, goblet cell metaplasia, and structural damage [41–47]. We hypothesize that deletion of PI3K*γ* decreases lung damage through the reduction of neutrophilic inflammation and neutrophil-associated active elastase. Consistently, a recent study demonstrated that NE activity is increased at the surface of airway neutrophils in PI3K*γ* ^WT^/*β*ENaC-Tg mice and patients with CF [6] and that genetic deletion of NE results in a significant reduction of emphysema-like changes in PI3K*γ* ^WT^/*β*ENaC-Tg mice, suggesting that NE is implicated in emphysema associated with chronic neutrophilic airway inflammation* in vivo*.

Recently, selective PI3K*γ* inhibitors have been developed and investigated in different mouse models of chronic inflammation [48–51]. Therefore, we evaluated the efficacy of the PI3K*γ* inhibitor AS-605240 on airway inflammation in *β*ENaC-Tg mice; we decided to use AS-605240 for its well characterized* in vivo* profile of efficacy and selectivity, indicated by the so far largest number of reports of pharmacological PI3K*γ* inhibition in mice [48–53]. We showed that treatment with the PI3K*γ* inhibitor decreased the number of neutrophils in BALF of *β*ENaC-Tg mice, thus reproducing the effect observed with the genetic deletion of PI3K*γ*. Several technical problems limit the assessment of the increased PI3K*γ* activity in *β*ENaC mice; however, the findings that PI3K*γ* ^WT^/*β*ENaC-Tg inflamed lungs have more leukocytes than PI3K*γ* ^KO^/*β*ENaC-Tg controls are an indirect indication of increased PI3K*γ* activity in these mice. Taken together, our data demonstrate the biological efficacy of both genetic deletion and pharmacological inhibition of PI3K*γ* in reducing chronic neutrophilic inflammation in CF-like lung disease* in vivo*.

Whereas blockade of PI3K*γ* activity by small-molecule inhibitors may represent a valid approach to modulate excessive leukocyte accumulation in inflamed tissues where leukocyte recruitment is correlated with disease progression, on the other hand increased susceptibility to infection might be a potential side effect of the use of these molecules. In this context, a previous study [54] showed that either gene deletion or pharmacologic inhibition of PI3K*γ* in mice infected with* S. pneumoniae* caused an impaired exudate macrophage recruitment associated with a reduced lung pneumococcal clearance and an impaired resolution/repair process, leading to progressive pneumococcal pneumonia. Thus, whereas pharmacological inhibition of PI3K*γ*, eventually in association with antibacterial treatment, may be a viable strategy to inhibit chronic inflammation and limit lung damage in stable CF lung disease, it might have adverse effects on host defense in acute infections when high bacterial burden occurs. In view of a clinical application of PI3K*γ* inhibitors, target validation will be an important future aspect to discriminate between specific effects of the drug and potential side effects.

## 5. Conclusions

Neutrophil-dominated airway inflammation has been implicated as a key feature of progressive lung damage in CF. Thus, reducing airway inflammation is a major goal to prevent lung damage and maintain lung function in CF. Current therapeutic strategies that aim to reduce chronic neutrophilic inflammation in the airways of CF patients have been largely unsuccessful. This study shows that genetic deletion and pharmacological inhibition of PI3K*γ* decrease neutrophilic airway inflammation and structural lung damage in a mouse model of CF lung disease. These results provide insight into the molecular mechanisms of chronic airway inflammation and suggest a novel treatment strategy to reduce inflammation and lung damage in patients with CF and potentially other neutrophilic airway diseases. Further studies with emerging PI3K*γ* inhibitors [49–51] are required to confirm the efficacy of these molecules and exclude their potentially adverse effects on host defense.
